# The kinked structure and interchain van der Waals interaction of carbyne nanocrystals†

**DOI:** 10.1039/d2sc04926k

**Published:** 2022-12-06

**Authors:** Weiwei Cao, Huakai Xu, Pu Liu, Yan He, Guowei Yang

**Affiliations:** a State Key Laboratory of Optoelectronic Materials and Technologies, Nanotechnology Research Center, School of Materials Science & Engineering, Sun Yat-sen University Guangzhou 510275 Guangdong P. R. China stsygw@mail.sysu.edu.cn; b College of Science, Guangdong University of Petrochemical Technology Maoming 525000 Guangdong P. R. China yanhe@gdupt.edu.cn

## Abstract

Carbyne with one-dimensional sp-hybridized carbon atoms is the third form of carbon following diamond and graphite. Although carbyne nanocrystals have been synthesized, little is known about its structural details. Here, we report experimental evidence of the kinked structure of carbon chains and interchain van der Waals interaction of carbyne nanocrystals by near edge X-ray absorption fine structure (NEXAFS) spectroscopy. We measure the 
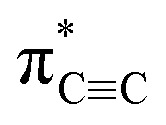
 resonance and the feature peaks of the kinked configuration of carbon chains and the van der Waals interaction between chains of carbyne nanocrystals using NEXAFS spectroscopy. We also perform theoretical calculations of density functional theory and simulations based on the super-cell core-hole method for carbon K-edge NEXAFS. The theoretical results are in good agreement with the experimental measurements, which demonstrates that carbyne nanocrystals are van der Waals crystals with kinked chains as structural units. Note that the peak at 288.5 eV in the simulated NEXAFS spectrum implies the possible presence of hydrogen-terminated kinks or hydrogen-terminated chains in carbyne nanocrystals, which clarifies the understanding of the C–H bond in carbyne nanocrystals. These findings are enlightening and significant for pursuing physics and potential applications of carbyne.

## Abstract 2

Carbyne with one-dimensional sp-hybridized carbon atoms is the third form of carbon following diamond and graphite.

## Graphical abstract

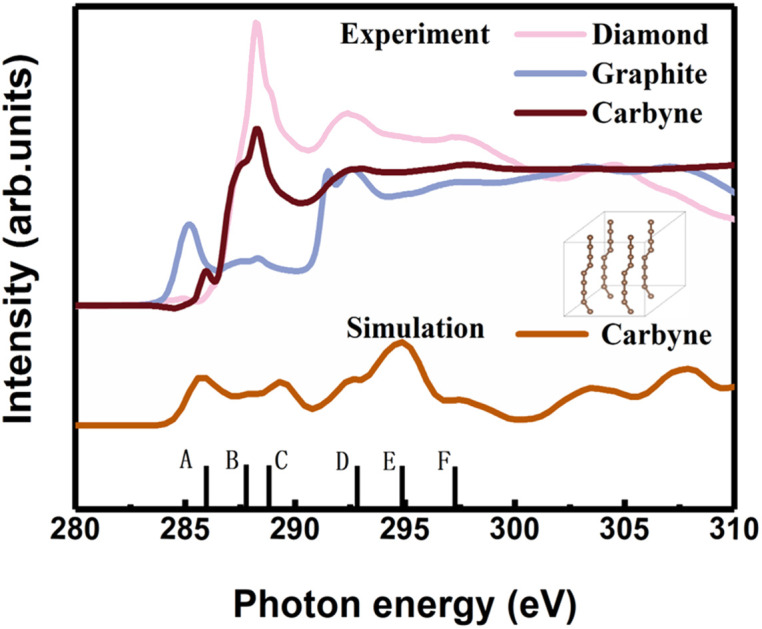

## Introduction

Carbon exists in various forms on the earth and in space, and is indispensable to all known animals and plants. Unique physical properties and different constructures of carbon have been found, studied, and applied. Carbon materials have a major demand in many industrial fields.^1–3^ According to the hybridization form of carbon atoms, carbon materials can be classified into three kinds in nature.^4^ The first form of carbon is represented by diamond and its derivatives like C_8_ (ref. 5) and lonsdaleite^6^ with three-dimensional sp^3^-hybridization. The second form of carbon is graphite and its derivatives including carbon nanotubes and graphene with two-dimensional sp^2^-hybridization.^7,8^ Carbyne with one-dimensional sp-hybridization is the third form of carbon following diamond and graphite.^9^ Recently, Pan *et al.* reported the first synthesis of carbyne nanocrystals by laser ablation in liquids (LAL).^10^ Since then, a large number of studies on carbyne nanocrystals have emerged,^11–13^ which implies that a new era of carbyne science and technology is coming.^8^ Note that Babaev and Cuseva prepared a solid carbon film by pulsed plasma deposition of carbon clusters generated by a vacuum arc, and they called this solid film “linear-chained carbon (LCC)” because they believed that an energetic ion beam can destroy the sp^2^ or sp^3^ hybrid structures of carbon and make them into sp hybrids.^14^

However, there are two important issues in the crystalline structure of carbyne that have so far not been clarified experimentally. Heimann *et al.* proposed that the stabilization of carbon chains of carbyne crystals can be achieved by kink formation in the geometrical structure.^15–17^ Pan *et al.* deduced that the interaction between carbon chains of carbyne nanocrystals is van der Waals force.^9^ But, there are no direct pieces of experimental evidence for the kinked structure of carbon chains and interchain van der Waals interaction of carbyne crystals. Theoretically, the kinked configuration of chains and van der Waals interaction between chains are the important reasons for the possible magnetism and room-temperature superconductivity of carbyne.^8^

As is well known, near edge X-ray absorption fine structure (NEXAFS) spectroscopy can be used to characterize various bonds and local environments of carbonaceous molecules, such as on diamond, graphite, and amorphous carbons which typically exhibit π* and σ* resonances.^18–21^ Hence, carbon K-edge NEXAFS is particularly effective in identifying the electronic structure of carbon nanomaterials. It could be used to distinguish between sp-hybridization, sp^2^ hybridization, and sp^3^ hybridization of carbon atoms.^22^ Consequently, it is viable to identify the sp-hybridization of carbyne nanocrystals by using the carbon K-edge NEXAFS spectra and to obtain more detailed structural information.

In this contribution, we reported the NEXAFS spectra of carbyne nanocrystals. Interestingly, the 
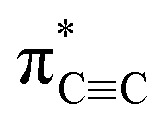
 resonance of carbyne was observed at 285.9 eV; meanwhile, two new peaks between the π* and σ* resonances were measured. By calculating and simulating carbon K-edge NEXAFS with different carbon nanostructures using density functional theory (DFT) and the super-cell core-hole method, respectively, we can attribute these two new peaks to the kinked structure of chains and interchain van der Waals interaction of carbyne nanocrystals. The peak at 288.5 eV in the simulated NEXAFS spectrum implies the possible presence of hydrogen-terminated kinks or hydrogen-terminated chains in carbyne nanocrystals, which clarifies the understanding of the C–H bond in carbyne nanocrystals. Therefore, this study clearly indicated that NEXAFS spectroscopy is an effective technique that can provide a more intuitive factual basis for structural information of carbyne nanocrystals.

## Results and discussion

**Fig. 1:**
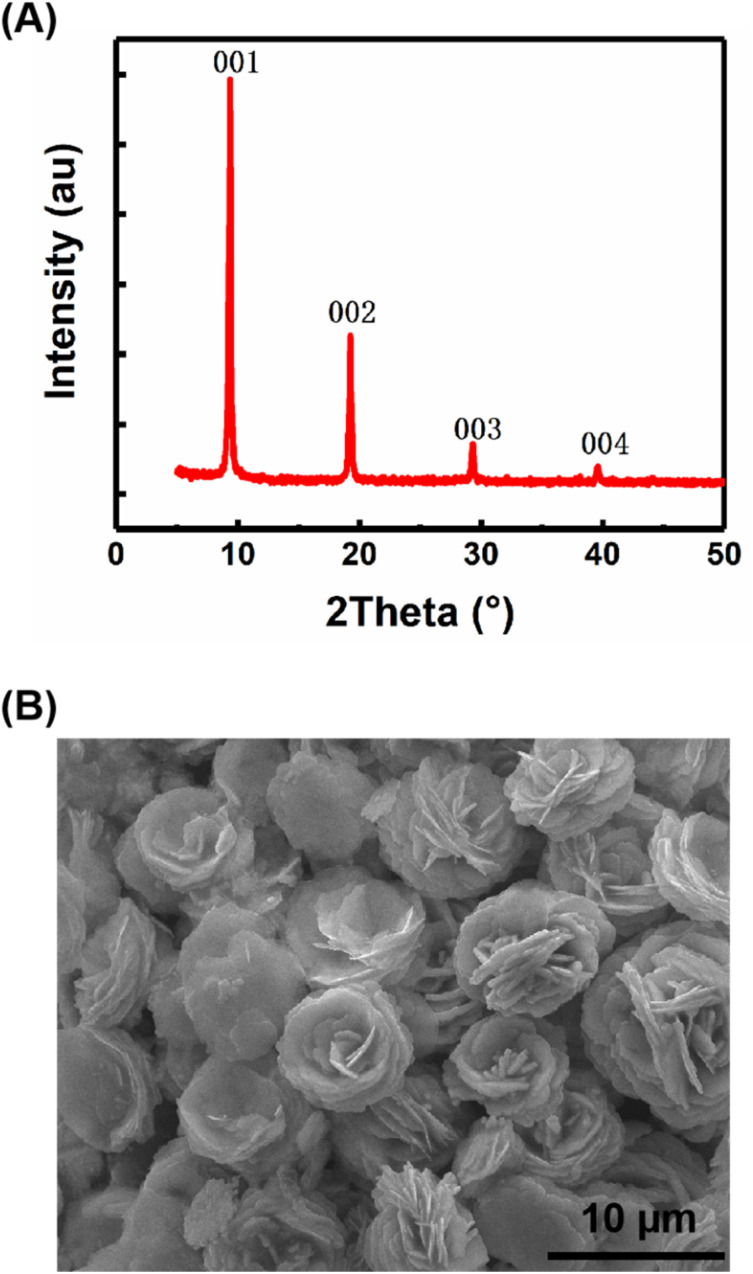
Structural characterization and morphology of carbyne nanocrystals, XRD pattern (A) and SEM image (B).

The samples of carbyne nanocrystals were prepared by the LAL technique. Fig. 1A shows the XRD pattern of the sample, which indicates that the sample has a hexagonal structure and displays four sharp and strong peaks (001), (002), (003), and (004). These results show a clear preference orientation along the *c*-axis. In addition, the sample was placed on a silicon wafer, which was analyzed and observed by using a scanning electron microscope, and the results clearly showed the stacked flake morphology (Fig. 1B). Notably, in our case, the samples of carbyne nanocrystals are very stable, and they are inert and do not decay or oxidize in air, just like diamond and graphite under normal conditions. Additionally, the XRD pattern was obtained to investigate the air stability of carbyne nanocrystals (Fig. S1 of the ESI†). Clearly, compared with the control group, the XRD pattern of that exposed in air for 60 days showed no observable changes in the position and intensity of each peak. Other detailed characterization studies, such as Raman spectra and transmission electron microscope images of carbyne nanocrystals can be referred to in the previous work.^10^

**Fig. 2:**
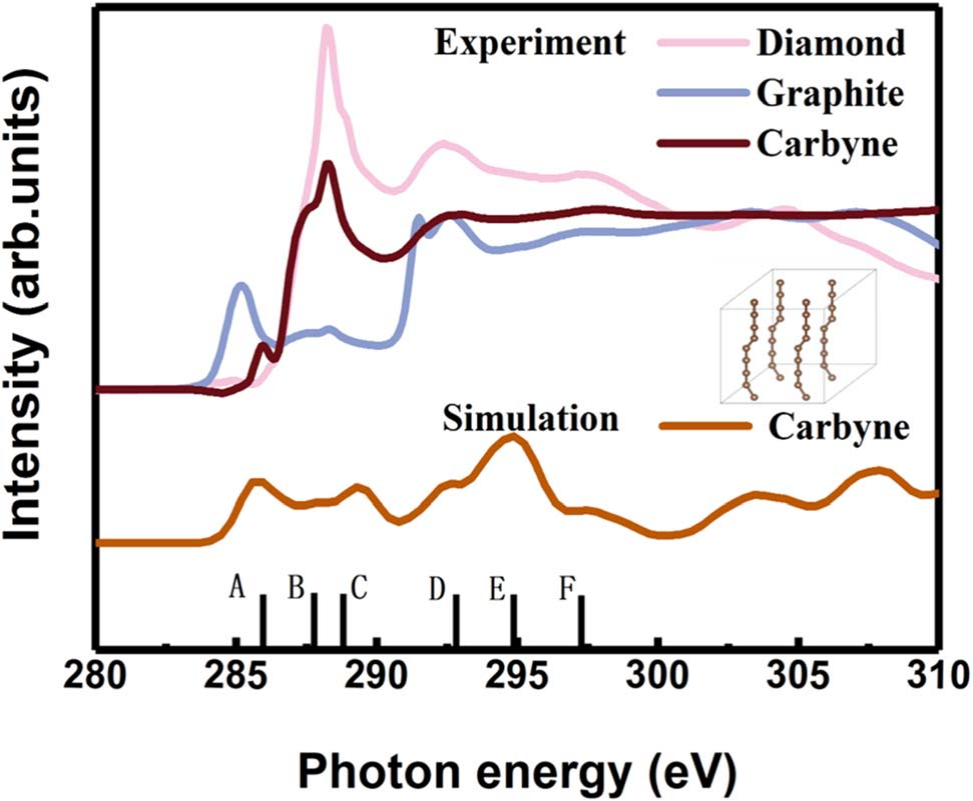
Experimental carbon K-edge NEXAFS spectra of carbyne nanocrystals, graphite, and diamond, as indicated on top with thick lines. The NEXAFS spectra predicted by DFT across the carbon 1s edge for the carbyne nanocrystals. Inset: the carbyne crystal structure model.

In order to clarify the detailed structural information of carbyne nanocrystals, the NEXAFS spectra of a carbyne nanocrystal, graphite, and diamond were compared together. It was obviously found that the peak shape of carbyne nanocrystals was distinctly different from that of graphite and diamond (Fig. 2). In addition, the NEXAFS spectra of the carbon 1s edge for a carbyne crystal, graphite, and diamond were predicted by DFT (Fig. 2 and S2 of the ESI†). Clearly, there are 5 peaks of the experimental spectrum with the energy change from 284 to 300 eV, whereas the multiple-scattering calculation shows 6 peaks. The energy of peaks A, B, C, D, E, and F are listed in Table 1. Although the intensity and broadening in our simulated NEXAFS are a little bit mismatched with the experimental results, the energy peaks (except for the E peak, discrepancy of the E peak between the experimental results and calculations will be explained in the following section) of theoretical predictions agree reasonably well with those of the experimental NEXAFS spectra except for the C peak (289.4 eV), which is ∼1 eV larger than that of experimental measurement. In fact, the results of intensity and broadening in the simulated NEXAFS of carbyne nanocrystals are slightly different from those of the experimental measurements, which is an unavoidable consequence of the VASP calculation. Because the observed broadening is driven by many factors depending on the particular experimental setup, it is not possible to reproduce the broadening exactly. Moreover, there are three possible reasons for the larger C energy peak in our calculation: (i) the influence of van der Waals interaction between carbyne chains. Since there is no reference for the chain’s van der Waals potential in the calculation, the potential energy between graphite layers is directly used in the calculation process. The experimental results show that the spacing between carbyne chains is about 0.334 nm,^10^ consistent with the spacing between graphite layers, which make the carbyne chain stable. Kudryavtsev *et al.* reported that an increase of the interchain interaction should lead to an instability of the chain structure, which may induce the chains collapsing into graphite or diamond.^23^ (ii) The influence of hydrogen atoms, which is unavoidable in the experimental process. The C–H bond will lead to the enhancement effect of the peak. These results have been confirmed by other studies.^24^ (iii) The influence of strain which is induced by the finite length of carbyne. As finite length carbyne is obtained in the laboratory, the strain will occur spontaneously, which will change the energy and bond length,^25^ and then affect NEXAFS. For instance, de Boer *et al.* reported the size dependent NEXAFS of carbon chains.^26^ Other contributions confirmed that strain has a significant effect on NEXAFS of nanostructures.^27,28^ Although the intensity and broadening in the simulation are slightly different from those measured in the experiment, the energy peaks between them are consistent, which indicates that the structure shown in the inset of Fig. 2 is a valid model for dealing with the relevant properties of carbyne crystals.

**Table 1:** The peak energies of carbyne crystal, graphite and diamond NEXAFS (in eV)^a^

Peak	A	B	C	D	E	F
Carbyne crystal*	285.9	287.7	289.4	292.7	294.9	297.4
Carbyne crystal^#^	285.9	287.5	288.3	292.8		297.7
Graphite*	285.5		288.3	291.9	295.6	297.3
				293		
Graphite^#^	285.5		288.3	291.6		297.0
				292.6		
Diamond*			288.2	292.0	295.9	297.3
Diamond^#^			288.2	292.2		297.2

a The superscripts * and # denote the calculations and experimental measurements, respectively.

**Fig. 3:**
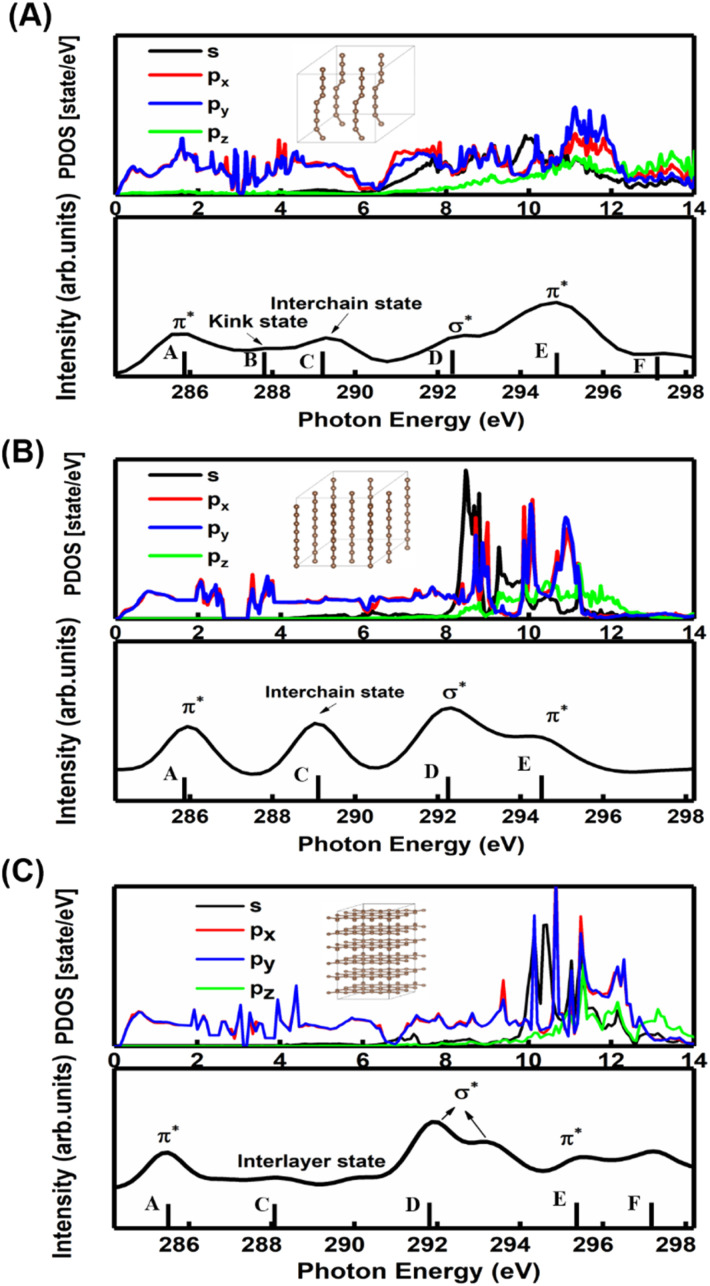
Comparison of the site-projected PDOS with the NEXAFS spectrum of the kinked carbyne crystal in (A), straight carbyne crystal in (B) and graphite in (C). Inset: Kinked carbyne crystal structure model, straight carbyne crystal structure model and graphite structure model.

A comparison between the site-projected partial density of states (PDOS) and the NEXAFS spectrum of the carbyne crystal is shown in Fig. 3A. The absorption edge in the carbyne crystal sample at ∼285.9 eV (A peak) can be assigned to the out-of-plane 1s → π* transition due to the overlapped p_*x*_ and p_*y*_ orbitals. The absorption edge at ∼292.7 (D peak) can be assigned to the in-plane σ* resonance as the number of states in the s orbital increases to overlap with the p_*z*_ orbital. The calculated energies of the distance between A and D resonance peaks are about 6.7 and 6.6 eV for the carbyne crystal and graphite, and they align well with those from the measured electron yield and earlier results.^29^ Compared with those of graphite and diamond (π* at 285.5 and σ* at 291.9 and 293.0 eV for graphite, and σ* at 288.2 and 292.0 eV for diamond), the A and D peak energies of the carbyne crystal (285.9 and 292.7 eV) are different. These results may relate to the discrepancy, the band structure and the transition process. The carbyne crystal is composed of alternating single and triple bonds,^8^ in which the sp-hybridized and non-hybridized p-orbitals form large numbers of π* bonds between carbon atoms, and a high state density peak is formed near the low conduction band.

Further evidence for these two peaks is the charge density distribution of the carbyne nanocrystal, as shown in Fig. S3A of the ESI,† and the charge is mostly distributed in the in-plane direction. These results indicated that the orbitals are slightly pointing at each other, which corresponds to the σ* bond in the D peak. Fig. S3B of the ESI† shows that the charge of the A peak is predominantly distributed in the out-of-plane direction, indicating that the p orbitals are parallel to each other, which corresponds to the π* bond. Therefore, we confirmed the existence of carbon–carbon triple bonds in the carbyne nanocrystal using NEXAFS. The previous Raman characterization of carbyne nanocrystals also showed that carbyne nanocrystals are formed by the sp hybridization of carbon atoms.^10^ Additionally, it has been shown that the peak at 285.9 eV is due to sp hybridization.^30^ To the best of our knowledge, this is the first NEXAFS spectrum of the carbyne nanocrystal displaying a π* peak indicative of sp hybridization.

In order to understand the broadening of B, C, E and F peaks in the carbyne crystal and graphite, Fig. 3B and C show the DOS of the carbyne crystal with a straight carbon chain and graphite, respectively. Obviously, the B peak vanishes in these two structures, while both the energy and intensity of the C, E and F peaks are similar to that of kinked structures of the carbyne crystal. On comparing PDOS between Fig. 3A and B, we can find that the E peak is due to the π* resonance which is formed by the highly overlapped p_*x*_ and p_*y*_ orbitals, and the F peak is due to the σ* resonance formed by the s and p orbitals. From PDOS of graphite in Fig. 3C, there are two π* resonance peaks (285.5 and 295.5 eV) and two σ* resonance peaks (292.5 and 297.8 eV). Note that the disappeared E peak in the experimental spectrum may be attributed to the incident angle and the weak π* resonance. The calculations agree well with other reports,^29,31^ and agree reasonably well with photo absorption studies of polycrystalline graphite and secondary-electron-emission work on crystalline graphite. In order to study the C peak, some reports indicated that this peak of graphite is a new kind of conduction state,^32,33^ the interlayer states, which exhibit a free-electron character parallel to the layers and relatively large charge densities outside the basal planes at ∼288 eV. Coincidentally, the C peak in carbyne nanocrystals is similar to that of the interlayer state as both states are caused by van der Waals interaction. Therefore, these calculated results (Fig. 3A and B) indicated that a new state would exist at ∼289 eV in the carbyne crystal with a kinked structure.

**Fig. 4:**
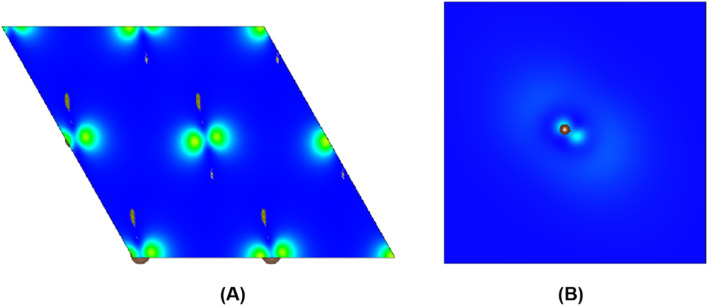
The charge density distribution between kinked carbon chains in (A) and the single kinked carbon chain in (B).

To further study whether there is a similar state to the interlayer state in the carbyne crystal, the charge density between carbon chains is shown in Fig. 4. We studied two carbyne system models: a carbyne crystal (Fig. 4A) and carbyne chain (Fig. 4B). Both the charge densities of these two structures are symmetric and their existence is independent of the distance between the chains. Its charge density shows that it is an interchain state which is not localized to the carbon atoms but exhibits a free-electron character in the planes parallel to the chains. Moreover, from Fig. 4B, the existence of charge density suggests that the interchain state also arises in a single carbyne chain. This result is similar to that of graphene, which likewise exhibits the interlayer state identical to that of graphite at ∼289 eV,^34^ which indicated that the C peak in carbyne nanocrystals is induced by the van der Waals interaction between chains. Moreover, by comparing the intensity of C peaks in Fig. 2, it was found that the experimental result of carbyne nanocrystals is much higher than that of the simulation. This result may be due to the influence of the π* resonance and different carbon species. According to our previous work, the synthesized carbyne nanocrystals contain C–H bonds.^10^ Since the sample was synthesized by LAL in ethanol, sp chains with terminal hydrogen are usually a common outcome.^35,36^ Based on this consideration, two models of hydrogen-terminated at the end and kink in carbyne nanocrystals (Fig. S4A and S4B of the ESI†) were proposed to study the influence of C–H bonds on the C peak. These results suggested that an extra peak at 288.5 appeared in those two models of hydrogen-terminated (Fig. S4C of the ESI†). Thus, these results are consistent with those reported in earlier studies.^24,27,37^ Moreover, it was found that the intensity of the C peak in the hydrogen-terminated kink strongly increases. Therefore, we believe that the hydrogen-termination plays a crucial role in modulating the potential energy between chains and disturbing the charge density. Importantly, this result is consistent with the experimental results with a strong peak at 288.3 eV (Fig. 2). Consequently, it is reasonable to believe that hydrogen-termination in carbyne nanocrystals is reasonable to retain the stability.

**Fig. 5:**
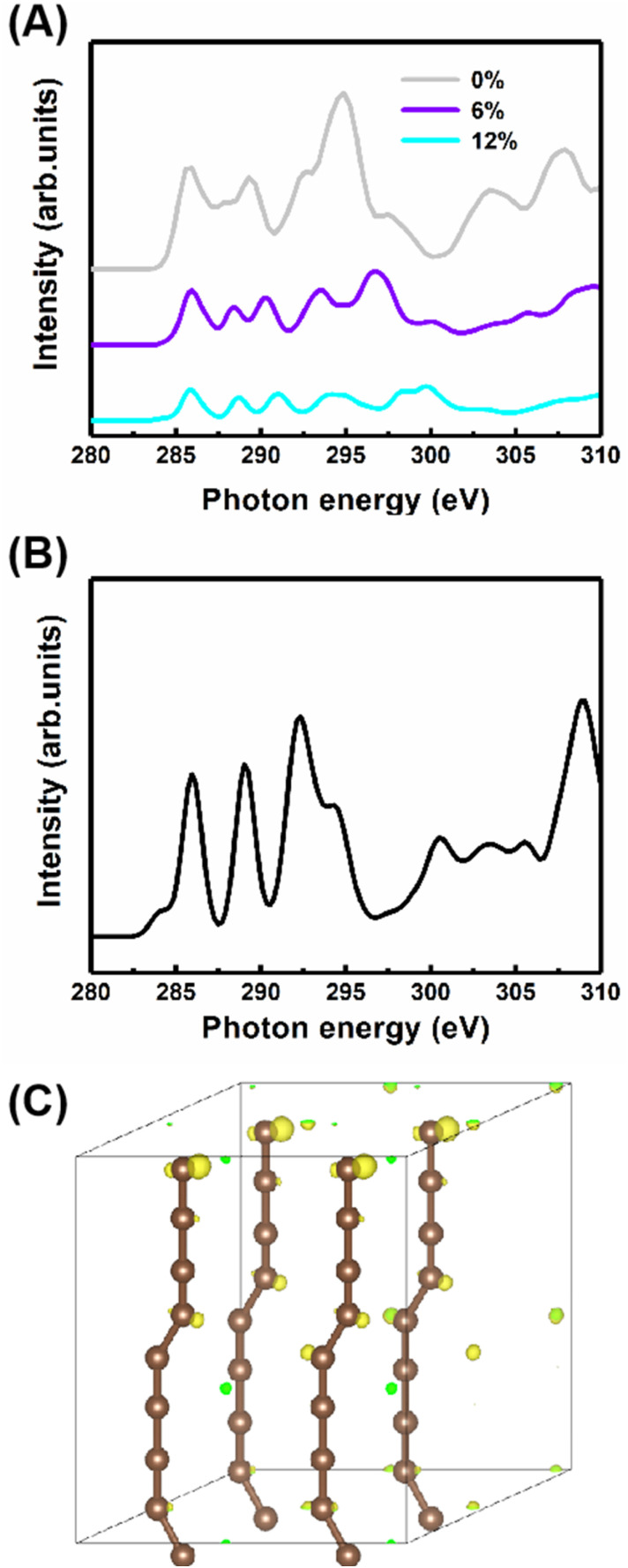
(A) Strain-dependent NEXAFS of the carbyne nanocrystals with kinked chains. (B) The NEXAFS spectra of carbyne nanocrystals with straight chains. (C) The charge density distribution in kinked carbon bonds.

Additionally, to study the characteristics of the B peak, the NEXAFS spectra of the carbyne crystal with the compressive strains of 0%, 6% and 12% are shown in Fig. 5A. All peaks show strain-dependent intensity and energy. Therefore, these results can be simply explained by the total electron yield signal being proportional to the photo absorption coefficient and band structure. Note that this result has been reported by earlier contributions.^29,38^ Importantly, the most dramatic result is the increase in the intensity of peak B at 287.7 eV as the strain increases, suggesting that peak B intensity increases with the kinking angle. Moreover, further calculations demonstrate that when the kinking angle tends to 0, this peak virtually disappears, as shown in Fig. 5B. Because of the angular dependence, the peak can be explicitly assigned to the kink state.

To further confirm the existence of the kinked structure, the charge densities of the carbyne crystal with the kinked carbon bonds are demonstrated in Fig. 5C. It is clearly seen that the kinked bonds induce the nonuniform distribution of charge density and it is mainly concentrated at the twisted carbon atoms, whereas the charge density of the carbyne crystal with straight chains is uniform as shown in Fig. S5 of the ESI.† Furthermore, the kinked structure of a carbyne chain and its charge density distribution is demonstrated in Fig. S6 of the ESI.† The nonuniform distribution of charge density induced by the kinked carbon bonds indicates that a kink state (B peak) arises at ∼288 eV, as shown in Fig. S7 of the ESI.† Interestingly, compared with the above results in Fig. S8 of the ESI,† the significant discrepancy in charge density between kinked and non-kinked carbyne structures lead to obvious differences in NEXAFS spectra, whereas since the van der Waals interactions between carbyne chains affect the charge density slightly, the NEXFAS spectra of kinked or non-kinked carbyne structures, carbyne nanocrystals and carbyne chains, are similar to each other. Additionally, to further clarify the nature of the energy shift of peaks B and C with increasing strain, Fig. S9 of the ESI† shows the total charge density of carbyne nanocrystals under various compressive strains, which suggests that the strain induces the spatial inhomogeneity of the chain orientation of charge density fluctuations and electronic properties. Clearly, the charge density in both the in-plane and out-of-plane directions increase with strain. For instance, the charge density is mainly distributed at kinked carbon atoms when the strain is fixed at 0%, whereas the charge density of the axial carbon atoms and interchains increases rapidly as the strain increases to 12%. Thus, these results suggest that B and C peaks may shift with the strain. Additionally, other reports indicated that the energy shift relates to strain which can modulate the electronic structure, Fermi level and charge density of nanostructures.^27,28,39,40^

## Conclusion

In summary, we have provided direct experimental evidence for the kinked structure of carbon chains and interchain van der Waals interaction of carbyne nanocrystals by NEXAFS spectroscopy. We have also performed the calculations and simulation of the carbon K-edge NEXAFS with different carbon nanostructures based on DFT and the super-cell core-hole method. The theoretical results are in good agreement with the experimental data, which demonstrated that carbyne nanocrystals are van der Waals crystals with kinked chains as structural units. The peak at 288.5 eV in the simulated NEXAFS spectrum implies the possible presence of hydrogen-terminated kinks or hydrogen-terminated chains in carbyne nanocrystals, which clarifies the understanding of the C–H bond in carbyne nanocrystals. These results will help scientists explore novel physical properties and potential technical applications of carbyne.

## Experimental

### Synthesis of carbyne nanocrystals

The synthesis of carbyne nanocrystals was based on the LAL method.^10^ First, a high-purity gold target (99.99% purity) was placed at the bottom of the reactor, and then 25 ml of alcohol was poured. The liquid level was about 8 mm above the gold target. Then, an adjustable second harmonic produced by using a Q-switched Nd:YAG laser device was used to focus on the gold target, with a wavelength of 532 nm, a pulse width of 10 ns, and a laser energy of 600 mJ per pulse. The laser focus spot size was about 1 mm. During the experiment, nitrogen must be introduced to protect the alcohol from burning. After approximately 25 minutes of pulsed laser action, the solution containing the CNC_S_ was collected. Then, the solution was purified by high-performance liquid chromatography (HPLC). Notably, carbyne nanocrystals were directly synthesized in the LAL possess. HPLC was employed to remove impurities such as amorphous carbon produced in the synthesis process. The purified samples were collected for subsequent experimental testing.

### Characterization of carbyne nanocrystals

X-ray diffraction (a Rigaku D/Max-IIIA X-ray diffractometer with Cu Kα radiation, at a scanning rate of 2° s^−1^) was used to characterize the crystal structure of carbyne nanocrystals. The morphology of the sample was observed using a scanning electron microscopy system (FEI Quanta-400). The NEXAFS spectra of the samples were collected at the Singapore Synchrotron Light Source (SSLS) center, where a pair of channel-cut Si (111) crystals was used in a monochromator. The energy resolution was set at ∼0.1 eV for C K edges. The K-edge absorption data are collected in total electron yield (TEY) mode monitoring the total current. The base pressure in the UHV chamber is maintained at ∼2 × 10^−10^ mbar throughout the measurements.

## Calculation and simulations

All DFT calculations were performed with the Vienna *ab initio* simulation package (VASP) software. To account for the van der Waals interactions between carbyne chains, we also adopted the DFT-D2 function in the carbyne crystal. There are 15 Å vacuum layers in the *x* and *y* directions of carbyne chain configurations to avoid artificial interaction between chains. The electronic plane wave interception energy was set to be 500 eV; the simulation was used to approximate exchange and correlation functions with the generalized gradient approximation (GGA),^41^ the Perdew–Burke–Ernzerhof (PBE) functional, and the convergence threshold for total energy was set to 10^−5^ eV. For integration in the Brillouin zone, a Monkhorst–Pack k-mesh of (6 × 6 × 3) was used for carbyne crystal calculations, and a 1 × 1 ×3 k-mesh was used for carbyne chain calculations.

The super-cell core-hole method is used for the simulation of the carbon K-edge NEXAFS spectra.^42,43^ The X-ray absorption cross sections are given by using
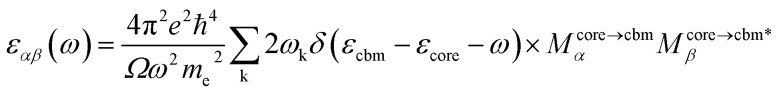
where *M* and *ε* denote momentum matrix elements and orbital energies. *M*^core→cbm^_*α*_ is the transition amplitude between the initial core state with energy *ε*_core_ and the all-electron (empty) final state, in the presence of a core hole on the absorbing atom with energy *ε*_cbm_. Here, we consider excitations only between the valence and conduction bands. The components of the dielectric tensor are indexed by using the Cartesian indices *α* and *β*. *Ω*, *e* and *m*_e_ denote the unit cell volume, electron chargef and mass of the electron, respectively. Since only one core hole at a single site is considered in the X-ray absorption spectrum, the momentum matrix elements from the core level to the conduction band can be obtained as follows:
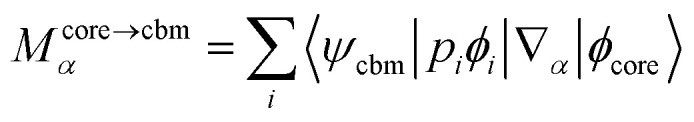


The |*ψ*〉 is the all-electron orbitals in the PAW method. The |*ϕ*〉 and |*p*_*i*_〉 are all-electron pseudo partial waves and the projectors, respectively. The index *i* is a shorthand for the atomic site and other indices enumerating these quantities at each site.

## Author contributions

W. W. C.: experimental work. H. K. X.: calculations. P. L.: data analysis. Y. H.: theoretical analysis. G. W. Y.: project planning.

## Conflicts of interest

The authors declare that they have no competing interests.

## Supplementary Material

SC-014-D2SC04926K-s001
