# Supplementary material for: The kinked structure and interchain van der Waals interaction of carbyne nanocrystals
Source: Chem Sci. 2022 Dec 6;14(2):338–44. doi: 10.1039/d2sc04926k (PMC9811524; doi:10.1039/d2sc04926k)
Supplement: SC-014-D2SC04926K-s001 [file SC-014-D2SC04926K-s001.pdf]

Electronic Supplementary Material (ESI) for Chemical Science.
This journal is © The Royal Society of Chemistry 2022

Supporting Information for

Kinked structure and interchain van der Waals interaction of carbyne nanocrystals

Weiwei Cao[1†], Huakai Xu[2†], Pu Liu, Yan He[2*]& Guowei Yang[1*]

[1] State Key Laboratory of Optoelectronic Materials and Technologies, Nanotechnology Research Center, School of Materials Science & Engineering, Sun Yat-sen University, Guangzhou 510275, Guangdong, P. R. China

[2] College of Science, Guangdong University of Petrochemical Technology, Maoming, 525000, Guangdong, P. R. China

†These authors contributed equally to this work.

*Corresponding authors: yanhe@gdupt.edu.cn, stsygw@mail.sysu.edu.cn

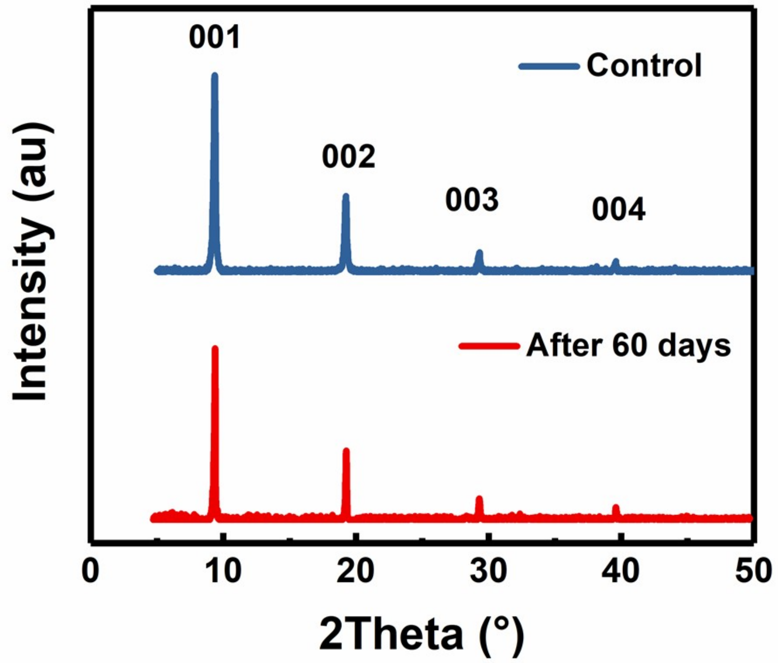

Figure S1. XRD of as-grown carbyne nanocrystals and after 60 days exposed in air.

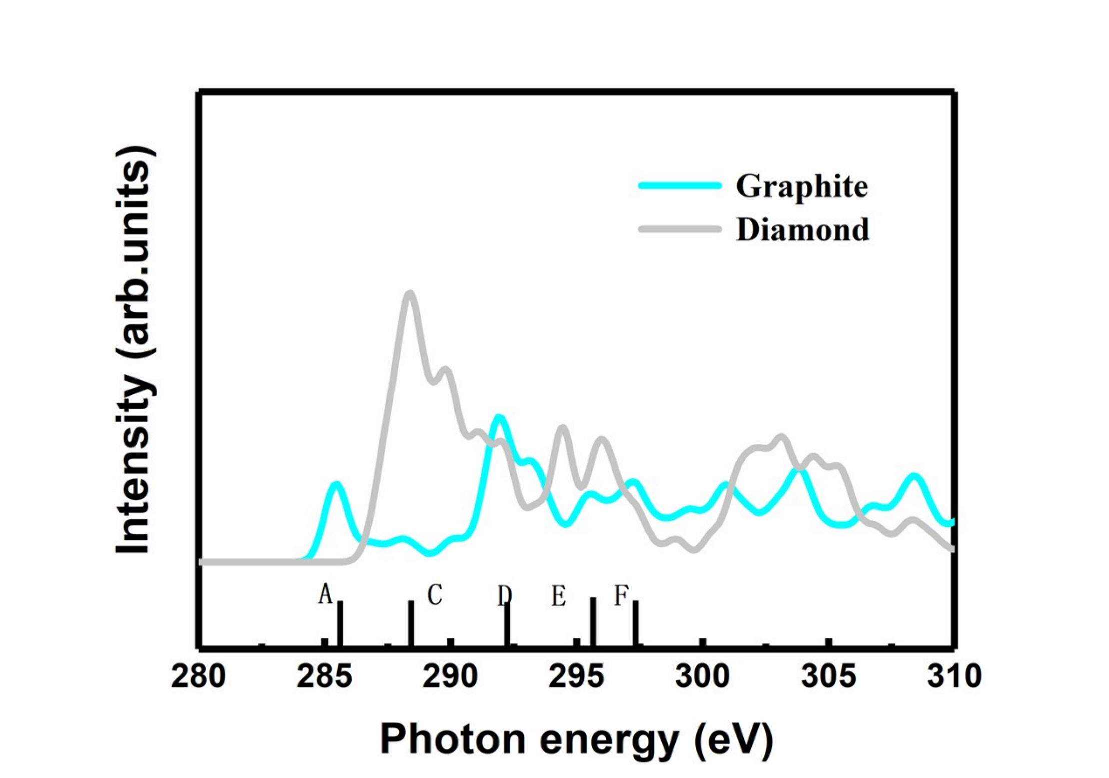

Figure S2. The NEXAFS spectra predicted by DFT across the carbon 1s edge for graphite and diamond.

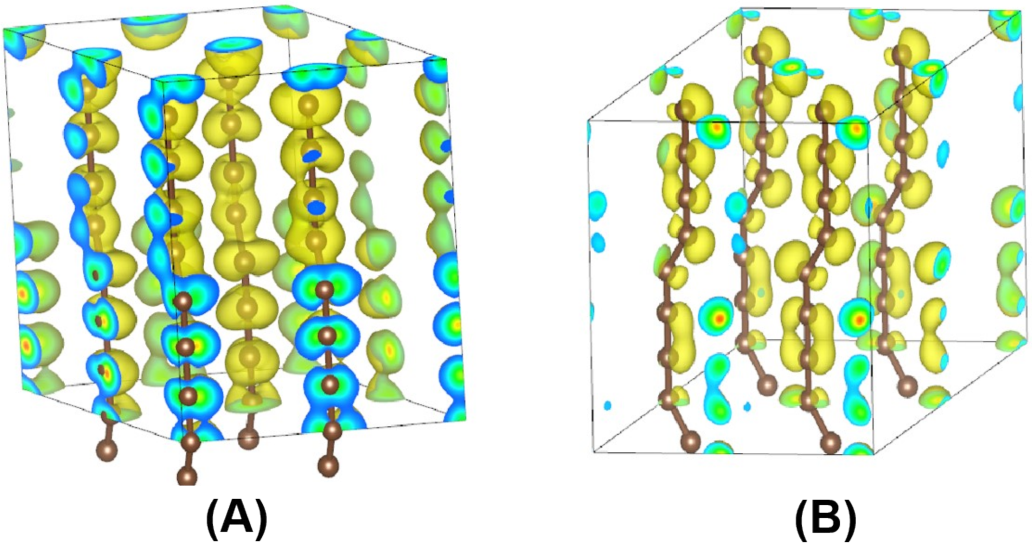

Figure S3. The charge densities distribution of π^* and σ^* bond in the carbyne crystal. The charge densities distribution: For (A), the charge is mainly distributed in the in-plane direction. This result suggests the orbitals are kind of pointing at each other, which corresponding to the σ^* bond. For (B), the charge is mainly distributed in the out-of-plane direction suggest the *p* orbitals are parallel to each other, which corresponding to the π^* bond.

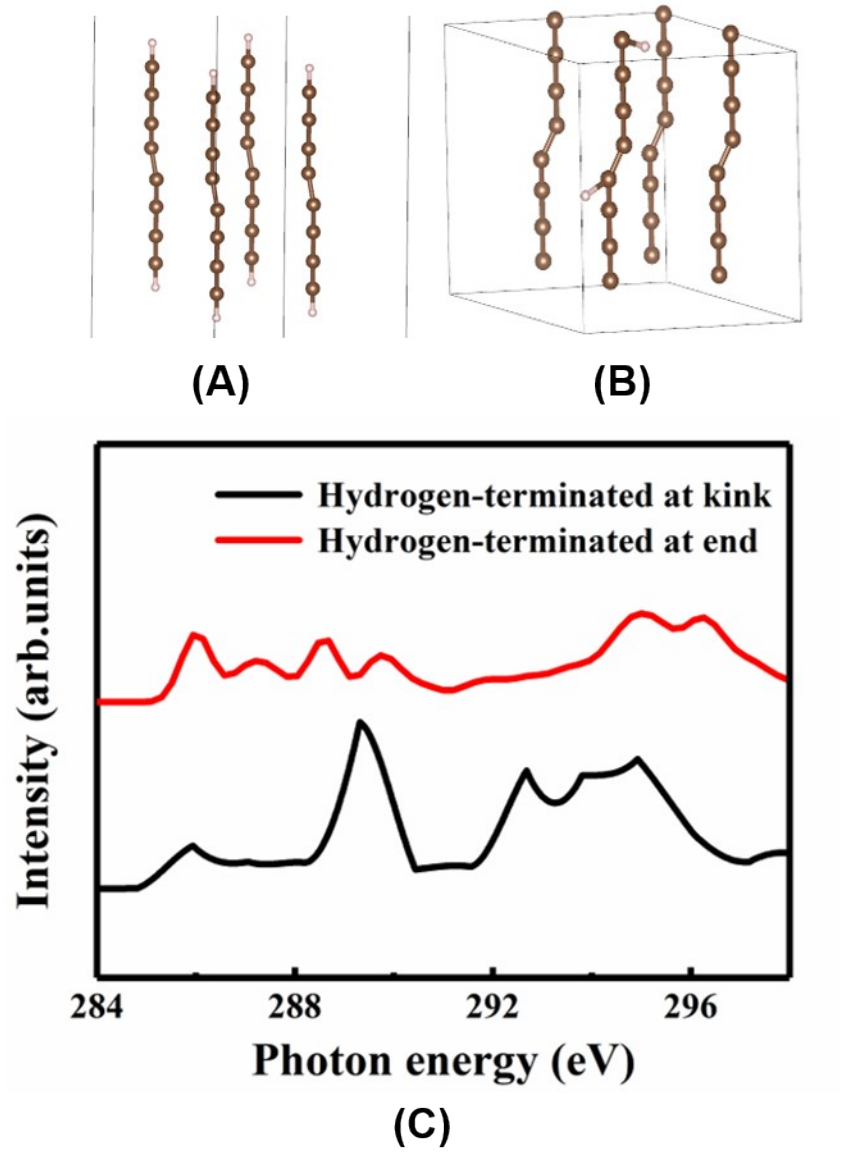

Figure S4. Hydrogen-terminated of carbyne nanocrystals. (A) hydrogen-terminated at end and (B) hydrogen-terminated at kink. (C) The NEXAFS spectrum of those two structures. An extra peak at ~288.5 is appeared in those two models of hydrogen-terminated, and the intensity of 289 peak in hydrogen-terminated kink become strongly increase.

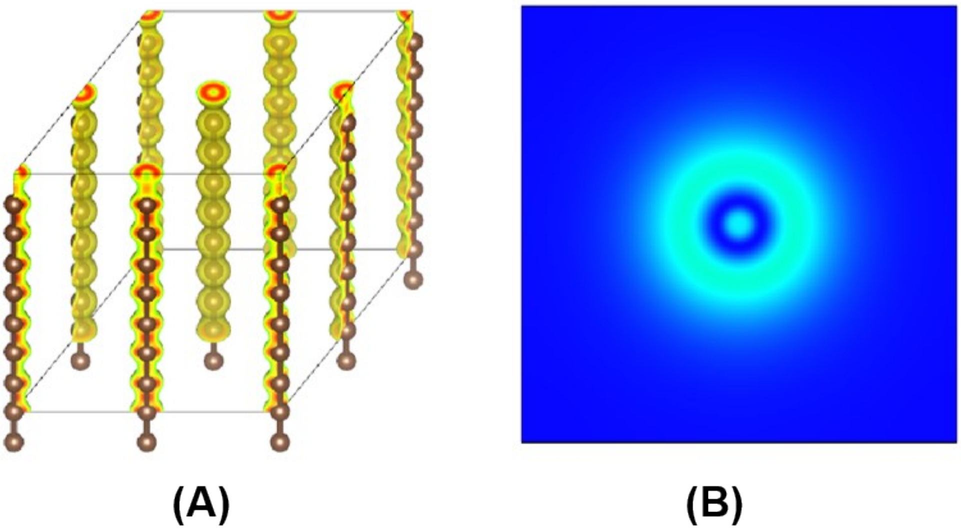

Figure S5. The charge densities distribution of the carbyne crystal. The charge densities distribution: (A) the charge density distribution of straight carbyne crystal, it is uniform; (B) the charge density distribution of straight carbyne chain, it is uniform.

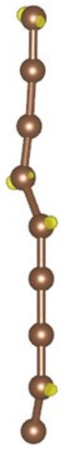

Figure S6. The charge densities distribution of a kinked carbyne chain. The charge densities distribution: the charge density is nonuniform. The kink bonds induce the nonuniform distribution of charge density and it is mainly concentrated at the twisted carbon atoms.

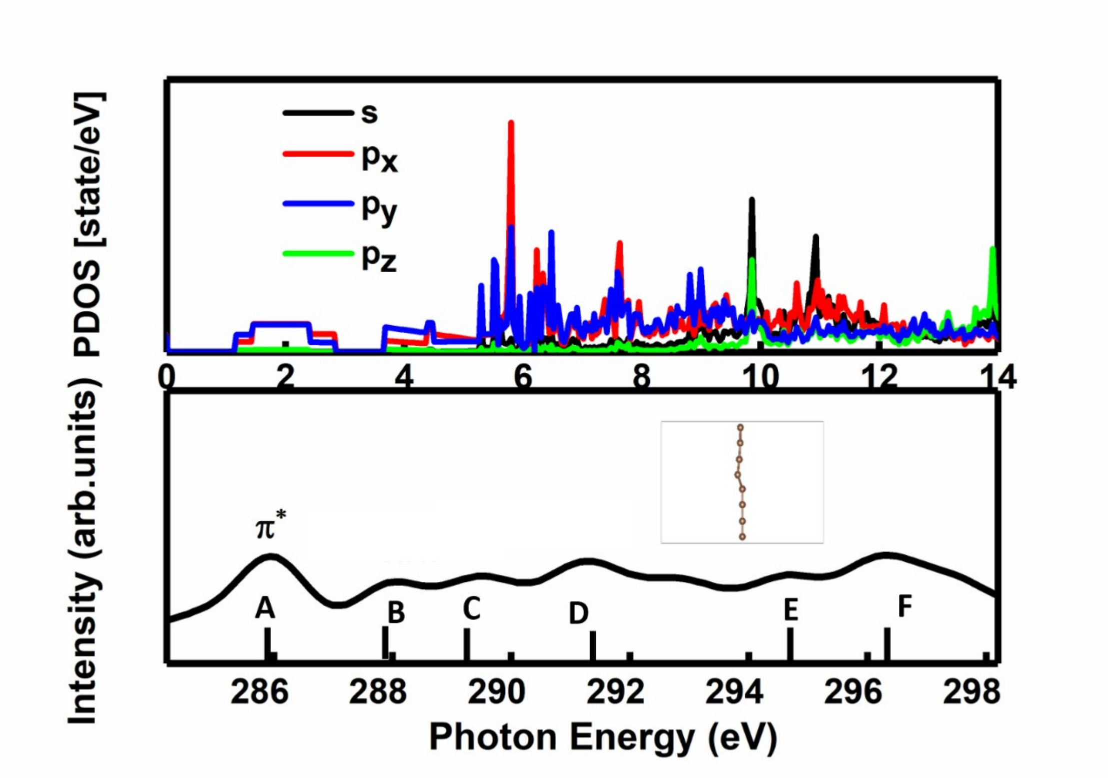

Figure S7. PDOS of the kinked carbon chain. PDOS and intensity of the kinked carbon chain: the kink state and interchain state are corresponding to the absorption edge at ~288 and ~289.5 eV.

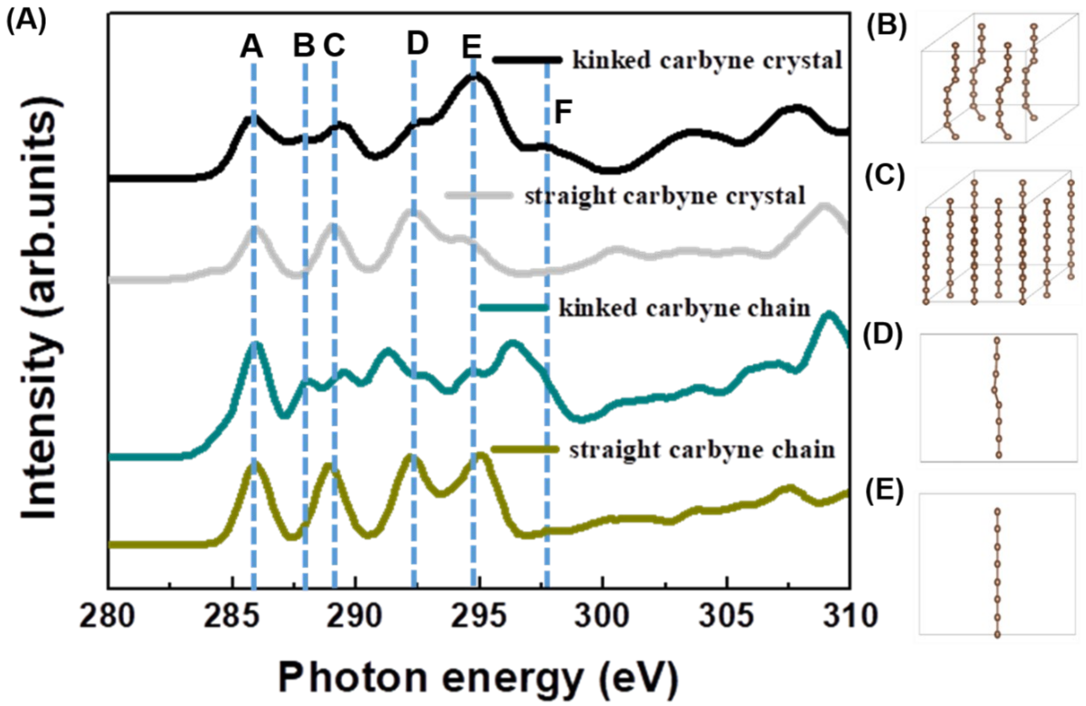

Figure S8. The NEXAFS predicted of carbyne. (A) The NEXAFS of kinked carbyne crystal, straight carbyne crystal, kinked carbyne chain, and straight carbyne chain. The peak at energy of ~288 eV is virtually disappeared for straight carbyne crystal and straight carbon chain, whereas it is arisen in the kinked carbyne crystal and chain. (B) Kinked carbyne crystal structure model. (C) Straight carbyne crystal structure model. (D) Kinked carbyne chain structure model. (E) straight carbyne chain structure model.

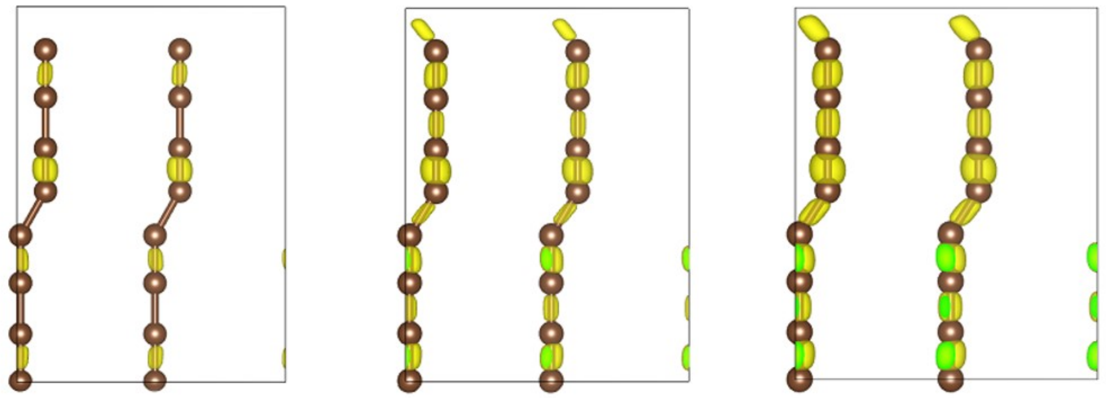

Figure S9. The total charge density distribution of carbyne nanocrystals under 0% (A), 6% (B), and 12% (C) strains. The charge density in both in-plane and out-of-plane directions increase with strain.
